# Pharmacist Prescribing as a Strategy to Facilitate Vaccine Accessibility in Ontario, Canada

**DOI:** 10.3390/pharmacy14060125

**Published:** 2026-08-25

**Authors:** Sherilyn K. D. Houle

**Affiliations:** School of Pharmacy, University of Waterloo, Waterloo, ON N2L 3G1, Canada; sherilyn.houle@uwaterloo.ca

**Keywords:** prescribing, vaccine, immunization

## Abstract

In Ontario, Canada, pharmacists and pharmacy technicians who have completed an approved injection training program are authorized to administer vaccines against 16 vaccine-preventable diseases, with other vaccines requiring an order from a physician or nurse practitioner authorizing their administration to a patient. Authorization to administer vaccines is separate from authorization to dispense vaccines. Some of these vaccines can be dispensed without a prescription, while others require a prescription for the pharmacist to be able to dispense the vaccine and/or to be able to bill the cost of the vaccine to a private insurance plan. Currently, Ontario pharmacists do not have authorization to prescribe any vaccines, whether it be for dispensing or insurance billing purposes. This article describes current practice in Ontario related to vaccine accessibility, summarizes vaccine-prescribing scope of practice adopted by other Canadian provinces and territories, and describes the potential benefits of expanding the pharmacist scope of practice in Ontario to include the prescribing of vaccines.

## 1. Introduction

Multiple factors impact the ability of residents of Ontario, Canada, to access vaccinations through community pharmacies. These include vaccine scheduling, pharmacy professional scope of practice, the public funding status of vaccines and pharmacy access to those publicly funded vaccines, and third-party insurance requirements. This article summarizes vaccine regulation and pharmacy scope of practice in Ontario and opportunities for modernization to support vaccine uptake.

### 1.1. Vaccine Scheduling

In Canada, the National Association of Pharmacy Regulatory Authorities (NAPRA) has a mandate to regulate the availability of products approved by Health Canada without conditions that the product be available only by prescription nationwide through the National Drug Schedules (NDS) program [1].

In the NDS review process, products are tested against criteria from most restricted to least restricted in a cascading approach [2], concluding with classification of a product into one of four categories [3]:

- Schedule I: Requires a prescription and can be provided to the public by a pharmacist following the diagnosis and professional intervention of a prescriber.
- Schedule II: Does not require a prescription but does require professional intervention by a pharmacist at the point of sale. These products must be physically contained within the pharmacy with no opportunity for patient self-selection.
- Schedule III: Available without a prescription but must be sold from a self-selection area of the pharmacy that is under direct supervision of a pharmacist. The pharmacist must be available, accessible, and approachable for assistance.
- Unscheduled: Can be sold without professional supervision and may be sold from any retail outlet.

Ontario follows the NDS schedules; however, there are several complicating factors that make these schedules challenging to navigate:

- In addition to the cascading evaluation approach outlined above, vaccines in Canada can also be automatically moved from Schedule I to Schedule II if they require “special enhanced public access due to disease outbreaks” or if they are part of a routine immunization program in most/all provinces and territories [4]. Changing public policy and disease epidemiology over time means that a vaccine may move between schedules. The timing of this can be particularly confusing for new vaccines introduced to the market as each province/territory independently considers whether they will fund it. Once at least 7 of Canada’s 13 provinces and territories publicly fund a vaccine, it moves from Schedule I to II. The time between market approval and public funding by provinces ranges from a median of 557 days in Quebec to 1130 days in Nova Scotia [5].
- Some vaccines are scheduled broadly by vaccine-preventable disease (where all vaccines against that disease considered to be similarly scheduled), while others have differing schedules based on whether the vaccine is for pediatric or adult use (e.g., hepatitis B) or are product- and/or indication-specific (e.g., oral inactivated cholera vaccine when used for prophylaxis against traveler’s diarrhea due to enterotoxigenic *E. coli* is Schedule II, but its use for cholera would be Schedule I) [4].
- There is not a central location where patients or health professionals can effectively search the scheduling status of a given vaccine, as the NAPRA website is not programmed to be searchable by a vaccine’s trade name.

Confusion over the schedule status of specific vaccines and whether a prescription is required impacts both the health professionals providing patient care and those who wish to access vaccination. The potential for differences in interpretation of vaccine schedules can impact public trust in the delivery of health services and may also lead to differences in vaccine accessibility depending on each healthcare provider’s interpretation of vaccine scheduling.

### 1.2. Pharmacist Scope of Practice

While pharmacists in 8 of Canada’s 13 provinces and territories have some authority to prescribe vaccines (in some jurisdictions additional credentialing may be required), pharmacists within Ontario are among the 5 provinces/territories that do not hold any prescribing authority for vaccines [6]. However, it is possible for a pharmacist in Ontario to obtain a medical directive where prescribing authority is delegated to them by an authorized prescriber such as a physician or nurse practitioner [7]. While the number of medical directives in place between prescribers and pharmacists is not systematically tracked in Ontario, it is relatively rare and often held by pharmacists with a specialized practice such as those who operate a travel clinic.

In Ontario, pharmacists and pharmacy technicians with current authorization to administer injections can administer vaccines against a defined list of diseases without requiring an order from a prescriber as listed below. Unless otherwise stated, these vaccines can be administered to patients age 5 years and older [8]:Bacille Calmette-GuerinMeningococcal diseaseCOVID-19 (age ≥ 6 months)PertussisDiphtheriaPneumococcal disease*H. influenzae* type bRabiesHepatitis ARespiratory syncytial virusHepatitis BTetanusHerpes zosterTyphoidHuman papillomavirusVaricellaInfluenza (age ≥ 2 years)Yellow feverJapanese encephalitis


Vaccines against diseases not listed above (e.g., measles/mumps/rubella, polio, or chikungunya) require an order from a prescriber authorizing their administration to a specific patient.

### 1.3. Public Funding and Pharmacy Access to Publicly Funded Vaccines

Ontario’s routine immunization schedule lists vaccines that are publicly funded and the populations eligible for each vaccine [9]. Updates to coverage for vaccines against pneumococcal disease and respiratory syncytial virus (RSV) are published separately [10,11,12]. Vaccines can be publicly funded for routine use (where age is the only qualifying factor for eligibility) or for high-risk use (where a patient must have at least one of the specified risk factors to receive vaccination or to receive additional doses beyond those that are included in the routine program).

Prior to 1 July 2026, Ontario pharmacies were only able to administer publicly funded vaccines against COVID-19 and influenza. This has since expanded to include publicly funded vaccines against diphtheria, herpes zoster, pertussis, pneumococcal disease, RSV, and tetanus for those meeting eligibility criteria [8]. Patients wishing to be vaccinated with a product that is not within the provincial immunization program or who do not meet eligibility criteria for publicly funded vaccine can purchase it privately. Patients who are eligible for a vaccine that is publicly funded for them but is not available through pharmacies can choose to either visit a medical clinic or public health office to access it at no charge or to purchase it privately through a pharmacy. Publicly funded vaccines and their availability through pharmacies is summarized in Table 1.

### 1.4. Third-Party Insurance Coverage and Vaccines

It is estimated that 24.6 million Canadians have private plan coverage for prescription drugs, and, in 2019, 66% of plan members were reimbursed at some level for vaccines [13,14]. A 2023 analysis found that across the ten largest group benefits providers in Canada, only four covered vaccines as a standard inclusion for all plan members with no maximums to coverage amounts. The remainder offered vaccine inclusion as an optional selection for plan sponsors or offered vaccines as standard coverage but with an annual maximum benefit of less than CAD 500 [15].

Nearly all insurance plans in Canada require patients to have a prescription for any vaccine claimed by a plan member, even those vaccines not requiring a prescription according to NAPRA vaccine scheduling. However, with an estimated 2.5 million Ontarians not having a regular family doctor [16], this requirement creates additional financial and accessibility barriers impacting the uptake of vaccines, even for those with an insurance policy that offers some coverage for vaccine costs.

## 2. The Patient Journey to Access Vaccines in Ontario

**Figure 1:**
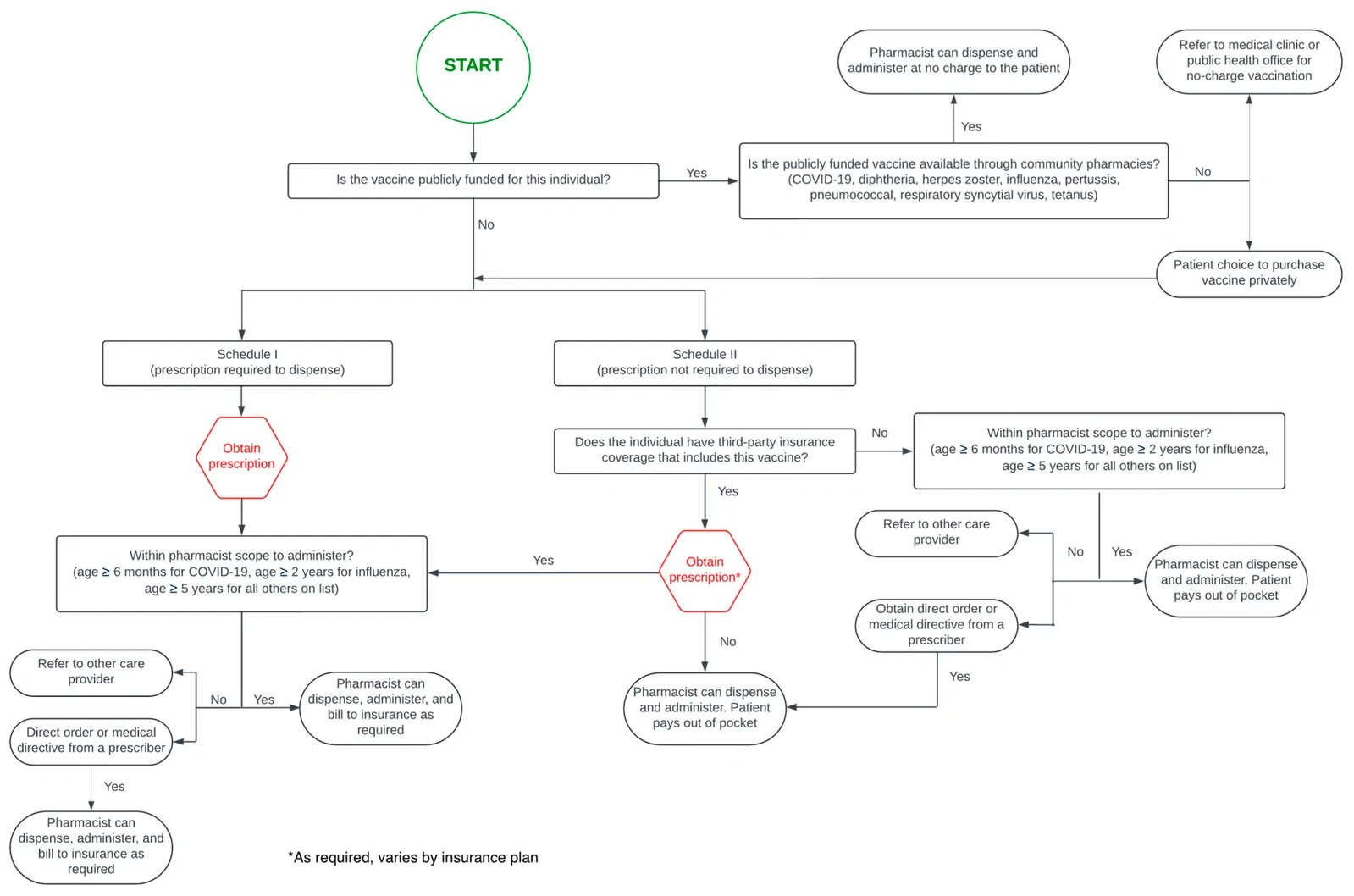
Current patient journey to access vaccines in Ontario, Canada.

Figure 1 illustrates the patient journey to access vaccines in Ontario, incorporating considerations around vaccine scheduling, pharmacist scope of practice, public funding of vaccines, pharmacy access to publicly funded vaccine, and insurance coverage as discussed above.

## 3. Patient Preferences, Knowledge, and Current Ability to Access Vaccinations

A 2021 survey of over 9000 Canadians found that Canadians valued flexible community-based options for accessing routine immunizations [17]:

- Firstly, 82% of respondents felt it is somewhat or very important that a vaccination site be close to their home and that it offer flexible booking options for appointments;
- Secondly, 76% felt it is somewhat or very important that a vaccination site offer walk-in services;
- Thirdly, 64% felt it is somewhat or very important that they be able to receive more than one vaccine at a time at a vaccination site.

The survey also found that Canadians are receptive to pharmacist-recommended vaccinations [17]:

- Firstly, 62% of respondents would take a pharmacist-recommended vaccine following a review of their records, with an additional 24% being unsure;
- Secondly, 56% of respondents would accept a routine immunization at a pharmacy even if they were at the pharmacy for another reason and the pharmacist offered to administer it, with an additional 27% being unsure.

Similarly, a 2025 survey of over 1500 residents of Ontario with a focus on human papillomavirus (HPV) vaccination found low awareness of vaccine availability overall and a desire for greater availability of vaccination at pharmacies [18]:

- Firstly, 32% reported being unsure of how adults in Ontario can access the HPV vaccine;
- Secondly, 76% want the HPV vaccine to be available at pharmacies without a prescription (only 12% were aware that this is currently in place);
- Thirdly, 76% believed that pharmacists should be able to prescribe all vaccines.

Publicly funded vaccines that are not available through pharmacies can be accessed through medical clinics or public health offices; however, accessibility to these providers may be challenging for some patients. For example, while 84% of Ontarians reported having a regular family physician in September 2023, 670,000 lived at least 51 km and more than 130,000 lived more than 200 km from their physician [16]. Operating hours for these providers may also be more limited than pharmacies. Publicly available information posted online identified that a very small number of public health offices in Ontario offer evening appointments, and none advertise regular weekend appointments; however, it is recognized that occasional clinic days may be held during high-demand periods and that mobile vaccination services are also occasionally offered [19].

In contrast, 76.4% of Ontarians living in a large urban center, 42.4% of those living in other urban centers, and 20.8% of those living in rural areas reside within 1 km of a pharmacy [20]. When this catchment area is extended to 5 km, these proportions increase to 99.4% of urban residents and 40.9% of rural residents; however, differences in accessibility vary depending on the geographic location of the center (northern vs. southern Ontario) and distance of rural communities from larger centers [21]. Pharmacies also often offer extended operating hours including evenings and weekends and the availability of walk-in services. Research from 2022 found that 89% of Ontario’s community pharmacies have operating hours on Saturdays and 50% on Sundays [20].

Research on appointments for injections scheduled at community pharmacies across Canada (inclusive of both vaccines and injectable medications) between January and December 2023 found that 27.0% of the over 1.2 million injections included in the analysis were scheduled during hours when other providers are less likely to be accessible, including evenings, weekends, and holidays [22]. Younger individuals (age < 18 years vs. 18–49, OR 1.74, 95%CI 1.69, 1.79) and males (vs. females, OR 1.05, 95%CI 1.03, 1.07) were significantly more likely to schedule appointments during these times, and these appointments were also significantly less likely to be booked at pharmacies located in rural centers (vs. urban centers, OR 0.77, 95%CI 0.75, 0.80), likely reflecting the greater availability of larger chain pharmacies with extended operating hours in urban centers [22]. It is important to note that injections sought by patients on a walk-in basis were not included in this analysis.

Access barriers were also found to impact vaccine uptake following comprehensive vaccination reviews by community pharmacists in Ontario [23]. In this study, 123 adult patients received an assessment guided by the VaxCheck clinical decision aid tool. The tool consolidated patient risk factors, current guidelines and vaccine availability, public coverage criteria, prescription requirements, and pharmacist scope of practice to identify unmet vaccination needs and generate an action plan to facilitate vaccination. Patients were largely willing to be vaccinated with the recommended vaccines that required a prescription to be dispensed or that were not publicly funded via pharmacies but had to be referred to other providers or delay vaccination until a prescription could be obtained. Overall, 50% of those recommended HPV, 39% recommended herpes zoster, and 32% recommended pneumococcal conjugate vaccination had to be referred to other settings for publicly funded vaccination, while 50% of those recommended meningococcal B, 35% recommended hepatitis A, 25% recommended hepatitis B, 19% recommended pneumococcal conjugate, and 12.5% recommended herpes zoster vaccination had to obtain a prescription before they could be vaccinated [23]. These referrals and delays may result in missed vaccination opportunities overall or create a potentially preventable period of susceptibility to vaccine-preventable diseases while awaiting access to the vaccine.

Telehealth and virtual visits are potentially viable options for those with physical accessibility barriers or lack of a regular primary care provider; however, service availability is inconsistent across providers. Some virtual care services are covered as part of Ontario’s publicly funded healthcare system, while others are hosted by private companies that charge users a fee per visit. Additionally, appointments may not be immediately available, creating a delay in accessing care.

## 4. Models of Pharmacist Prescribing and Scheduling of Vaccines Across Canada

Currently, 9 of Canada’s 13 provinces and territories have enacted legislation around vaccine scheduling in their jurisdiction that may differ from NAPRA schedules and/or authorize various models of pharmacist prescribing of vaccines as summarized in Table 2.

The policies described in Table 2 may involve prescribing authorization for NAPRA Schedule II (non-prescription) vaccines or may allow the dispensing of NAPRA Schedule I (prescription) vaccines without a prescription; regardless, a pharmacist’s clinical assessment for safety and appropriateness is required for all vaccines dispensed and/or administered. While there have been calls for harmonization of both routine immunization schedules [38,39] and pharmacist scope of practice nationwide [40,41,42,43], notable differences remain. Coordination of immunization schedules and programs remains a key pillar to address within Canada’s National Immunization Strategy for 2025–2030 [44].

Other models of pharmacist prescribing of vaccines have been implemented internationally; however, these will not be summarized here. Readers are encouraged to consult publications from the International Pharmaceutical Federation (FIP) for additional information on international scope of practice [45,46].

## 5. A Simplified Patient Journey with an Expansion to Pharmacists’ Scope

**Figure 2:**
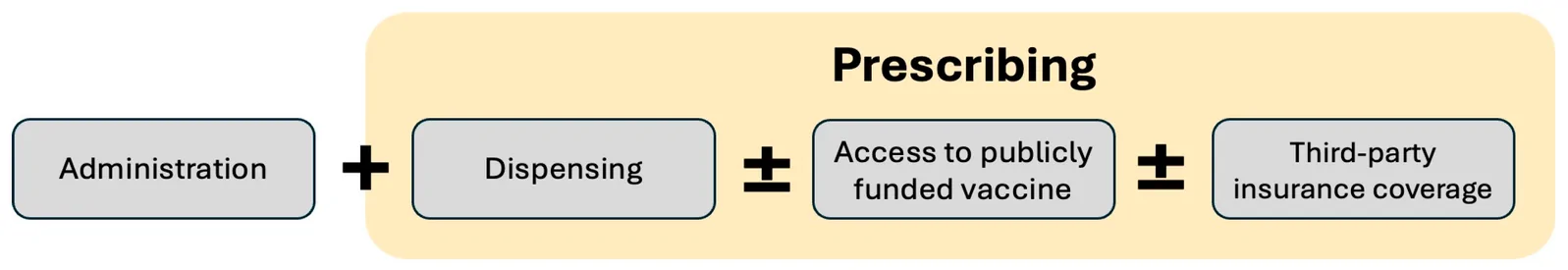
Policy-related barriers to vaccination and those impacted by pharmacist prescribing.

To be most effective, vaccination programs in Canada must simultaneously consider four policy-related barriers: (1) vaccine administration; (2) vaccine dispensing (i.e., scheduling); (3) access to publicly funded vaccine; and (4) third-party insurance coverage. Of these, dispensing, access to publicly funded vaccine, and third-party insurance coverage can be influenced by pharmacist scope of practice to prescribe vaccines (Figure 2).

Different models of pharmacist prescribing and vaccine scheduling exist across Canada, with varying impacts on the policy-related barriers. Saskatchewan and Quebec offer the broadest prescribing authority, with Saskatchewan’s only restriction being the prescribing of some travel vaccines only by pharmacists holding the ISTM Certificate in Travel Health. While Alberta authorizes the prescribing of all vaccines, this scope is restricted to pharmacists who have successfully obtained Additional Prescribing Authorization and is therefore not universal. Finally, the British Columbia approach of considering any vaccines recommended by an established provincial, national, or international association to be non-prescription and is therefore very broad and addresses dispensing-related barriers; however, lack of prescribing authority for pharmacists means that insurance-related barriers to coverage persist.

**Figure 3:**
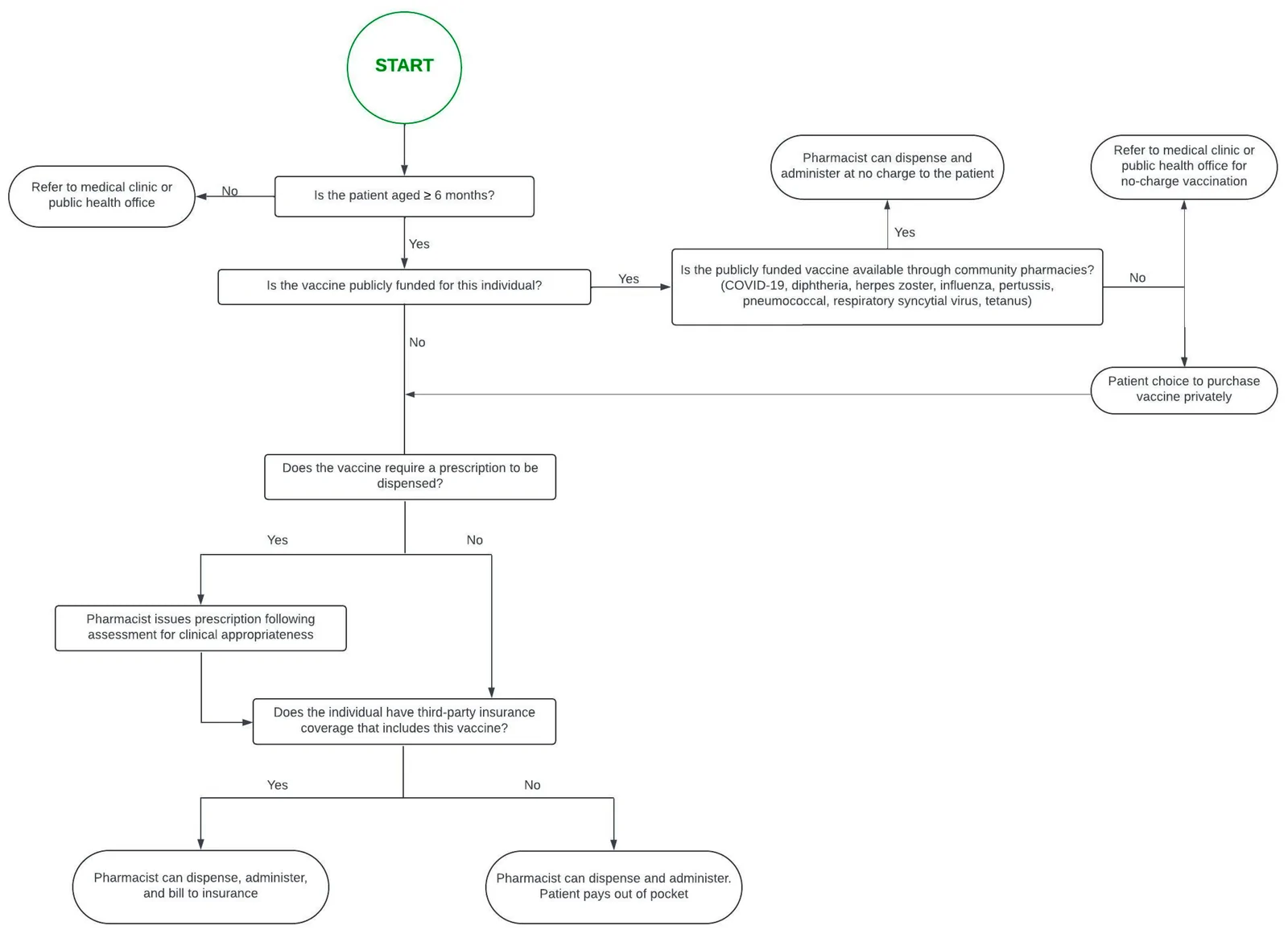
Patient journey to access vaccines in Ontario, Canada, if pharmacist prescribing of vaccines were enacted and minimum patient age to administer vaccines was consistently set at age ≥ 6 months for all vaccines (currently only applicable to COVID-19 vaccines).

If pharmacist prescribing was broadly implemented in Ontario, neither of the two “Obtain prescription” barriers in the patient journey to access vaccination in Figure 1 would remain. However, complexity would still exist related to access to publicly funded vaccine supply and pharmacist scope of practice to administer vaccines to children. If expansions to scope of practice for both pharmacist prescribing and the ability to administer any vaccine to patients ≥ 6 months of age (as is currently in place for COVID-19 vaccine), the patient journey would be notably simplified as illustrated in Figure 3. The only remaining reasons for referral to another healthcare provider would now be for the administration of a publicly funded vaccine not available through community pharmacies or vaccination for an infant < 6 months of age.

Pharmacists have also expressed willingness to expand their scope of practice related to vaccination and their ability to proactively offer vaccination reviews and additional vaccine administration services. While these activities are recognized to impact workload, it is seen as an important service to the community that they are willing to embrace if adequately supported to [47,48].

## 6. Discussion

Scope of practice expansions to authorize pharmacists to issue prescriptions for vaccines and to apply the minimum patient age of ≥6 months for administration as currently in place for COVID-19 vaccine to all vaccines can significantly simplify vaccine accessibility for Ontarians. As the dispensing of any vaccine (regardless of being Schedule I or Schedule II) requires an assessment of clinical appropriateness by a pharmacist, the ability to issue a prescription meets an administrative requirement without a significant impact on workload. Workload may in fact be simplified by reducing the need to request and track the receipt of prescriptions from other prescribers and can reduce confusion related to a vaccine’s NAPRA scheduling since a prescription can be issued for a vaccine regardless of its scheduling. Similarly, a prescription can be issued regardless of a patient’s insurance plan requirements to ensure any claims made to the plan are eligible for coverage if vaccine coverage is offered.

## 7. Conclusions

Ontario’s vaccination programming can be made less complex for both patients and health professionals through expansions in scope of practice for pharmacists related to vaccine administration and prescribing and enhanced pharmacy access to publicly funded vaccines. To support advocacy efforts, a series of three evidence briefs outlining current challenges and potential opportunities that can be realized from pharmacist prescribing of vaccines in Ontario are provided as Supplementary Materials (Figures S1–S3) and may serve as templates for other jurisdictions striving for similar expansions in scope.

## 8. Future Directions

Modernization of Ontario’s vaccination programs is ongoing and requires a coordinated effort between pharmacy regulators (to expand scope of practice around administering and prescribing vaccines), pharmacy advocacy organizations (to ensure remuneration for services is sufficient to offset costs), and governments (to allow more publicly funded vaccines to be made available to pharmacies). Taken together, these efforts have the potential to meaningfully transform vaccination services in the province.

## Figures and Tables

**Table 1:** Public funding of vaccines in Ontario and their availability through pharmacies.

Vaccine	Routine Use	High-Risk Use	Pharmacy Access to Publicly Funded Supply
COVID-19	Yes	N/A	Yes
Diphtheria, tetanus, pertussis, polio, *H. influenzae* type b combined	Yes	Yes	No
Hepatitis A	No	Yes	No
Hepatitis B	Yes	Yes	No
*H. influenzae* type b	Yes	Yes	No
Herpes zoster	Yes	No	Yes
Human papillomavirus	Yes	Yes	No
Influenza	Yes	N/A	Yes
Polio	Yes	Yes	No
Meningococcal B	No	Yes	No
Meningococcal C	Yes	No	No
Meningococcal ACYW	Yes	Yes	No
Measles, mumps, rubella combined	Yes	Yes	No
Measles, mumps, rubella, varicella combined	Yes	No	No
Pneumococcal	Yes	Yes	Yes
Respiratory syncytial virus	Yes	Yes	Yes
Rotavirus	Yes	No	No
Tetanus, diphtheria (with or without pertussis) combined	Yes	No	Yes
Tetanus, diphtheria, pertussis, polio combined	Yes	Yes	No
Varicella	Yes	Yes	No

N/A: Not applicable (funded for all with an indication for vaccination).

**Table 2:** Prescription status and pharmacist authority for the prescribing of vaccines in Canada.

Province, Year Implemented	Policy Description
Alberta, 2007 [24,25]	Pharmacists with Additional Prescribing Authorization (APA) can prescribe any vaccine In 2025, 66% of all pharmacists in Alberta had APA
British Columbia, 2009 [26]	All publicly funded vaccines can be dispensed without a prescription, as well as any vaccination recommended by any recognizable provincial, national, or organizational organization No prescribing authority for vaccines
Manitoba, 2014 [27]	No prescription required for HPV, pneumococcal, Tdap, or Td vaccines when provided to eligible people within the provincial publicly funded immunization program No prescribing authorization for other vaccines or for patients ineligible for publicly funded vaccines
New Brunswick, 2014 [28,29]	Levels of pharmacist prescribing: Section 1 vaccines (all pharmacists): Cholera (oral, inactivated vaccine only), diphtheria, Hib, hepatitis A, hepatitis B, HPV, herpes zoster, measles/mumps/rubella, meningococcal, pertussis, pneumococcal, polio, RSV, tetanus, traveler’s diarrhea, varicella Travel vaccines (ISTM Certificate in Travel Health required): Cholera (except oral, inactivated vaccine), European tick-borne encephalitis, Japanese encephalitis, rabies, typhoid, yellow fever
Newfoundland and Labrador, 2024 [30,31]	Pharmacists can prescribe vaccines for cholera (oral, inactivated vaccine only), diphtheria, Hib, hepatitis A, hepatitis B, herpes zoster, HPV, measles, mumps, rubella, meningococcus, pertussis, pneumococcus, polio, RSV, rotavirus, tetanus, varicella
Nova Scotia, 2011 [32]	Prescriptions not required for all vaccines in the Nova Scotia routine immunization schedule All pharmacists can prescribe hepatitis A, hepatitis B, herpes zoster, HPV, meningococcal, pneumococcal, typhoid, RSV, and varicella vaccines Pharmacists with the ISTM Certificate in Travel Health or a Diploma in Travel Medicine (Royal College of Physicians and Surgeons of Glasgow) can prescribe additional vaccines for the prevention of travel-related diseases
Prince Edward Island, 2020 [33,34,35]	Levels of pharmacist prescribing: Schedule A vaccines (all pharmacists authorized to administer injections): Diphtheria, hepatitis A, hepatitis B, herpes zoster, HPV, pertussis, pneumococcal, rabies, RSV, tetanus Schedule B vaccines (Pharmacists with accredited training on these diseases): European tick-borne encephalitis, Japanese encephalitis, typhoid, yellow fever
Quebec, 2019 [36]	Prescribing authority for all vaccines and all patients
Saskatchewan, 2019 [37]	All vaccines other than influenza require a prescription Levels of pharmacist prescribing: Non-travel vaccines (all pharmacists): Cholera (oral, inactivated vaccine only), diphtheria, Hib, hepatitis A, hepatitis B, herpes zoster, HPV, measles, mumps, rubella, pertussis, pneumococcal, polio, tetanus, varicella Travel vaccines (ISTM Certificate in Travel Health required): Cholera (except oral, inactivated vaccine), European tick-borne encephalitis, Japanese encephalitis, rabies, typhoid, yellow fever

APA: Additional Prescribing Authorization; Hib: *H. influenzae* type b; HPV: Human papillomavirus, ISTM: International Society of Travel Medicine; RSV: Respiratory syncytial virus; Td: Tetanus and diphtheria vaccine; Tdap: Tetanus, diphtheria, and acellular pertussis vaccine.

## Data Availability

No new data were created or analyzed in this study. Data sharing is not applicable to this article.

## Supplementary Materials

The following supporting information can be downloaded at https://www.mdpi.com/article/10.3390/pharmacy14060125/s1: Figure S1: Vaccine regulation and pharmacy professional scope of practice in Ontario; Figure S2: Vaccine accessibility from pharmacies: A Canadian environmental scan; Figure S3: The societal impact of pharmacist prescribing of vaccines.

## Abbreviations

The following abbreviations are used in this manuscript:

CAD	Canadian dollar
CI	Confidence interval
FIP	International Pharmaceutical Federation
Hib	*Haemophilus influenzae* type b
HPV	Human papillomavirus
ISTM	International Society of Travel Medicine
NAPRA	National Association of Pharmacy Regulatory Authorities
NDS	National Drug Schedules
OR	Odds ratio
RSV	Respiratory syncytial virus
Td	Tetanus and diphtheria vaccine
Tdap	Tetanus, diphtheria, and acellular pertussis vaccine

## References

[1] Drug Scheduling in Canada. https://www.napra.ca/national-drug-schedules/drug-scheduling-in-canada/.

[2] Summary of the National Drug Scheduling Process. https://www.napra.ca/national-drug-schedules/summary-of-the-national-drug-scheduling-process/.

[3] What Are the National Drug Schedules?. https://www.napra.ca/national-drug-schedules/what-are-the-national-drug-schedules/.

[4] Vaccines. https://www.napra.ca/nds/vaccines/.

[5] Time to Public Vaccine Access in Canada. https://www.iqvia.com/-/media/iqvia/pdfs/canada/publications/time-to-vaccine-access-en.pdf.

[6] Prescribing Authority of Pharmacists Across Canada. https://www.pharmacists.ca/cpha-ca/assets/File/pharmacy-in-canada/PharmacistPrescribingAuthority_EN_web.pdf.

[7] Medical Directives and the Delegation of Controlled Acts Policy. https://ocpinfo.com/pharmacy-professionals/rules-and-standards-of-the-profession/practice-policies-guidelines/medical-directives-and-the-delegation-of-controlled-acts/.

[8] Expanding Scope of Access—Vaccines. https://ocpinfo.com/wp-content/uploads/2026/05/expanding-scope-of-practice-vaccines.pdf.

[9] Publicly Funded Immunization Schedules for Ontario. https://www.ontario.ca/files/2024-01/moh-publicly-funded-immunization-schedule-en-2024-01-23.pdf.

[10] Health Care Provider Fact Sheet: Pneumococcal Conjugate Vaccines for Adults Aged 18 Years and Older. https://www.ontario.ca/files/2025-10/moh-hcp-fact-sheet-pneumococcal-vaccine-18-and-older-en-2025-10-03.pdf.

[11] Health Care Provider Fact Sheet: Pneumococcal Conjugate Vaccines for Children Aged 6 Weeks to 17 Years. https://www.ontario.ca/files/2025-07/moh-hcp-fact-sheet-pneumococcal-vaccine-6-weeks-to-17-years-en-2025-07-01.pdf.

[12] Respiratory Syncytial Virus (RSV) Prevention Programs. https://www.ontario.ca/page/respiratory-syncytial-virus-rsv-prevention-programs.

[13] Understanding the Gap 2.0: A Pan-Canadian Analysis of Prescription Drug Insurance Coverage. https://www.conferenceboard.ca/in-fact/understanding-the-gap/.

[14] The Plan Sponsor’s Guide to Vaccines. https://www.benefitscanada.com/microsite/the-plan-sponsors-guide-to-vaccines/.

[15] Private Drug Plan Leadership: Vaccine Coverage Report Card 2023. https://premium.hrreporter.com/ca-2023-exclusivefeature-private-drug-plan-leadership-vaccine-coverage-report-card-2023/p/1.

[16] New Data Shows There Are Now 2.5 Million Ontarians Without a Family Doctor. https://ontariofamilyphysicians.ca/news/new-data-shows-there-are-now-2-5-million-ontarians-without-a-family-doctor/.

[17] Routine Immunizations in Canada Following the COVID-19 Pandemic. https://neighbourhoodpharmacies.ca/sites/default/files/2021-10/Routine%20Immunization%20Final%20Results%20-%20Public%20Slides%20-%2020Oct2021%20vF_0.pdf.

[18] Most Ontarians (76%) Want Pharmacists to Prescribe HPV Vaccines, but Barriers Persist. https://fmwc.ca/hpv-vaccination-survey-most-ontarians-76-want-pharmacists-to-prescribe-hpv-vaccinations-but-barriers-exist/.

[19] Public Health Unit Locations. https://www.ontario.ca/page/public-health-unit-locations.

[20] Measuring Proximity to Services and Amenities: An Experimental Set of Indicators for Neighbourhoods and Localities. https://www150.statcan.gc.ca/n1/pub/18-001-x/18-001-x2020001-eng.htm.

[21] Timony P., Houle S.K.D., Gauthier A., Waite N.M. (2022). Geographic distribution of Ontario pharmacists: A focus on rural and northern communities. Can. Pharm. J..

[22] Houle S.K.D., Bhaidani S., Rehmanji T., Alsabbagh M.W. (2026). Administration of vaccines and injectable medications beyond 9 to 5: A cross-sectional analysis of over 1.2 million injection appointments scheduled at Canadian community pharmacies. J. Am. Pharm. Assoc..

[23] Houle S.K.D., Waite N.M., Vernon-Wilson E., Dolovich L., Yang M. (2024). Uptake and outcomes of VaxCheck, an adult life-course vaccination service: A study among community pharmacists. Vaccine.

[24] Standards of Practice for Pharmacists and Pharmacy Technicians. https://abpharmacy.ca/wp-content/uploads/Standards_SPPPT.pdf.

[25] Additional Prescribing Authorization Matters for Safe Pharmacy Practice in Alberta. https://abpharmacy.ca/news/additional-prescribing-authorization-matters-for-safe-pharmacy-practice-in-alberta/.

[26] Pharmacists and Publicly Funded Vaccines in B.C.: General Information. https://www2.gov.bc.ca/assets/gov/health/health-drug-coverage/pharmacare/vaccine-guide.pdf.

[27] Administration of Injections: Frequently Asked Questions. https://cphm.ca/wp-content/uploads/Resource-Library/Expanded-Scope/Administration-of-Injections-Reference-July-2025.pdf.

[28] Common Ailments and Certain Conditions. https://nbpharmacists.ca/en/page_category/legislation/.

[29] Travel Medicine: Prescribing for Preventable Diseases. https://nbpharmacists.ca/wp-content/uploads/2022/11/Travel-Medicine-_Prescribing-of-Restricted-Vaccines_Regs-Appendix3_2014-3.pdf.

[30] Standards of Practice: Prescribing by Pharmacists. https://cpnl.ca/media/SOP-Prescribing-by-Pharmacists-2023-10.pdf.

[31] Prescribing Standards of Practice. https://cpnl.ca/professional-practice/area-of-practice/prescribing/.

[32] Standards of Practice: Prescribing Drugs. https://nspharmacy.ca/wp-content/uploads/SOP_PrescribingDrugs_May2024.pdf.

[33] Regulated Health Professions Act: Pharmacist and Pharmacy Technician Regulations. https://www.princeedwardisland.ca/sites/default/files/legislation/r10-1-07-regulated_health_professions_act_pharmacist_and_pharmacy_technician_regulations.pdf.

[34] Application Requirements—Extended Practice Authorization in Drug Administration. https://pepharmacists.ca/wp-content/uploads/2025/08/RP07-Application-Requirements-EPC-DA-MAY-2025.pdf.

[35] Application Requirements—Extended Practice Certificate: Travel Vaccine Authority. https://pepharmacists.ca/wp-content/uploads/2023/11/RP10-Application-Requirements-EPC-Travel-Approved-Sept-2023.pdf.

[36] La Vaccination par le Pharmacien: Guide D’exercice. https://www.opq.org/wp-content/uploads/2020/03/Guide_vaccination_juil2023.pdf.

[37] Travel Health Services and Vaccine-Preventable Diseases FAQs. https://scp.in1touch.org/document/5581/REF_Travel_Health_FAQs.pdf.

[38] Macdonald N., Bortolussi R. (2011). A Harmonized Immunization Schedule for Canada: A Call to Action. Paediatr. Child Health.

[39] Toward Harmonized Adult Vaccination Schedules Across Canada. https://vaccines4life.com/mobilizing-policy-projects-and-studies/toward-harmonized-adult-vaccination-schedules-across-canada/.

[40] Emberley P. (2018). Canadian Pharmacists Scope 20/20: A vision for harmonized pharmacists’ scope in Canada. Can. Pharm. J..

[41] Pharmacists Scope of Practice: Neighbourhood Pharmacies’ Position. https://neighbourhoodpharmacies.ca/sites/default/files/2023-01/2022%20Pharmacists%20Scope%20of%20Practice%20Position%20Statement%202022%20V1.pdf.

[42] Raiche T., Pammett R., Dattani S., Dolovich L., Hamilton K., Kennie-Kaulbach N., McCarthy L., Jorgenson D. (2020). Community pharmacists’ evolving role in Canadian primary health care: A vision of harmonization in a patchwork system. Pharm. Pract..

[43] Fonseca J., Pearson-Sharpe J., Houle S., Waite N.M. (2019). Time for harmonization—Pharmacists as immunizers across Canadian jurisdictions. Can. Pharm. J..

[44] 2025–2030 Interim National Immunization Strategy. https://www.canada.ca/en/public-health/services/publications/vaccines-immunization/2025-2030-interim-national-immunization-strategy.html.

[45] Leveraging Pharmacy to Deliver Life-Course Vaccination: An FIP Global Intelligence Report. https://www.fip.org/file/5848.

[46] An Overview of Pharmacy’s Impact on Immunisation Coverage: A Global Survey. https://www.fip.org/file/4751.

[47] Waite N.M., Houle S.K.D., Toppari K., Pereira J.A. (2024). Willingness of Canadian community pharmacists to adopt a proactive life-course approach to vaccination services. J. Am. Pharm. Assoc..

[48] Vernon-Wilson E., Comrie M.L., Barrera K., Yang M., Dolovich L., Waite N.M., Houle S.K. (2025). Implementation of an adult life-course vaccine review service, VaxCheck, in community pharmacy: A qualitative analysis. J. Am. Pharm. Assoc..

