# Supplementary material for: Pharmacist Prescribing as a Strategy to Facilitate Vaccine Accessibility in Ontario, Canada
Source: Pharmacy (Basel). 2026 Aug 25;14(6):125. doi: 10.3390/pharmacy14060125 (PMC13610780; doi:10.3390/pharmacy14060125)
Supplement: Supplementary file 1 [file pharmacy-14-00125-s001.zip › Figure S1.pdf]

EVIDENCE BRIEF

Vaccine regulation and pharmacy professional scope of practice in Ontario

UNIVERSITY OF WATERLOO | SCHOOL OF PHARMACY

Vaccine accessibility in Ontario is complex. The public must navigate vaccine prescription status, the ability of pharmacists to administer the vaccine, public funding status and eligibility, and pharmacies' ability to access publicly funded vaccines for eligible Ontarians.

(see figure on reverse)

Vaccine prescription status process in Ontario[1-2]

New vaccine approved by Health Canada → **Schedule I** (available by prescription only) → **Schedule II** (available upon pharmacist consultation)

If any of the following are satisfied:
- Review by National Drug Scheduling Advisory Committee following manufacturer application
- Adopted as part of routine immunization program by ≥7 of 13 provinces/territories
- Outbreak (meningococcal vaccine)

- Prescription status can change over time and varies across vaccines (occasionally also by age)
- Third-party insurance plans often require a prescription to cover vaccines, even if Schedule II

Vaccine administration scope by pharmacists in Ontario[3]

List-based scope of practice: Ontario pharmacists can independently administer only a specified list of vaccines. Administration of vaccines outside this list requires an order from a prescriber to do so, regardless of the prescription status to dispense the vaccine

Inconsistent minimum patient age for pharmacist vaccine administration:

**6 months**<br>COVID-19 vaccine	**2 years**<br>Influenza vaccine	**5 years**<br>All other vaccines

Publicly funded vaccines and Ontario pharmacies

Ontario currently provides routine public funding for 18 vaccines (with some eligibility limitations), and funding for high-risk patients for 13 vaccines[4-5]

- As of July 1, 2026, pharmacies will be able to access **influenza, COVID-19, tetanus, diphtheria, pertussis, pneumococcal, respiratory syncytial virus (RSV) and shingles from the publicly funded supply** to offer to eligible patients.[6] Previously, only influenza and COVID-19 vaccines were accessible to pharmacies

Ontarians eligible for publicly funded vaccines **other than those specified above** can only access these at no charge from a medical clinic or public health unit. Those who are unable to access these settings or who wish to obtain it from their pharmacy must procure privately-purchased vaccine, which introduces inequity in accessibility related to ability to pay.

Vaccination Facts

341%

Return on investment (from averted healthcare costs and productivity gains) for every dollar spent on six vaccines* routinely used in adults[7]

65%

Proportion of Ontario adults who want more options to receive routine immunizations at their local pharmacy[8]

B-

Grade assigned by CanAge for the provision of adult vaccines for those aged 50+ in Ontario[9]

*Herpes zoster (shingles), RSV, pneumococcal, HPV, COVID-19, and influenza vaccines

This complexity results in confusion among Ontarians and their health professionals and contributes to missed opportunities to vaccinate. Suboptimal vaccination rates risk adverse impacts on individuals, populations, and societal productivity.

Funding for this project was provided by Merck Canada Inc. Merck Canada Inc. had no role in the development of the content, analysis, conclusions, or recommendations presented herein. The content reflects the independent views of the authors and is intended for informational purposes only.

EVIDENCE BRIEF

Vaccine regulation and pharmacy professional scope of practice in Ontario

UNIVERSITY OF WATERLOO | SCHOOL OF PHARMACY

The patient journey to access vaccinations through pharmacies in Ontario, other than for those eligible for publicly-funded vaccines available to pharmacies *

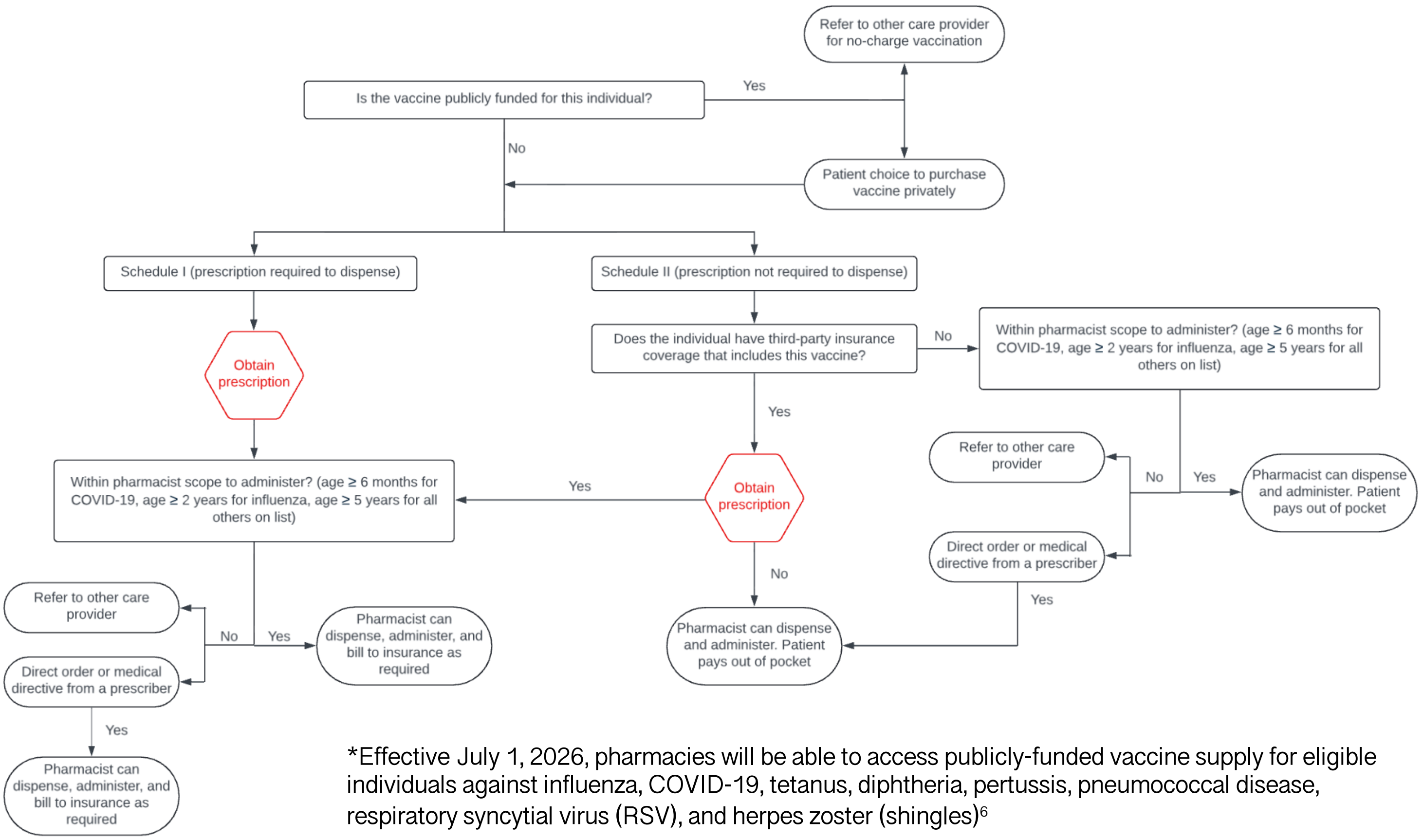

*Effective July 1, 2026, pharmacies will be able to access publicly-funded vaccine supply for eligible individuals against influenza, COVID-19, tetanus, diphtheria, pertussis, pneumococcal disease, respiratory syncytial virus (RSV), and herpes zoster (shingles)[6]

References:

1. **Drug Scheduling in Canada.** National Association of Pharmacy Regulatory Authorities. https://www.napra.ca/national-drug-schedules/drug-scheduling-in-canada/
2. **Vaccines.** National Association of Pharmacy Regulatory Authorities. https://www.napra.ca/nds/vaccines/
3. **Administering Injections.** Ontario College of Pharmacists. https://ocpinfo.com/wp-content/uploads/2025/05/administering-injections.pdf
4. **Publicly Funded Immunization Schedules for Ontario.** Ontario Ministry of Health. https://www.ontario.ca/files/2024-01/moh-publicly-funded-immunization-schedule-en-2024-01-23.pdf
5. **Respiratory Syncytial Virus (RSV) Prevention Programs.** Ontario Ministry of Health. https://www.ontario.ca/page/respiratory-syncytial-virus-rsv-prevention-programs
6. **Ontario Expanding Scope of Practice for Pharmacists and Other Health Professionals.** Ontario Ministry of Health. https://news.ontario.ca/en/release/1007431/ontario-expanding-scope-of-practice-for-pharmacists-and-other-health-professionals
7. **The Unmet Value of Vaccines in Canada.** Adult Vaccine Alliance. https://www.adultvaccinealliance.ca/
8. **Routine Immunizations in Canada Following the COVID-19 Pandemic.** Neighbourhood Pharmacy Association of Canada and 19toZero. https://neighbourhoodpharmacies.ca/sites/default/files/2021-10/Routine%20Immunization%20Final%20Results%20-%20Public%20Slides%20-%2020Oct2021%20vF_0.pdf
9. **Vaccine Report Card, 2025.** CanAge. https://canage.ca/advocacy/vaccine-report-card-2025/

Author

Sherilyn Houle, PhD
School of Pharmacy, University of Waterloo

https://uwaterloo.ca/pharmacy/profile/shoule

May 2026

Funding for this project was provided by Merck Canada Inc. Merck Canada Inc. had no role in the development of the content, analysis, conclusions, or recommendations presented herein. The content reflects the independent views of the authors and is intended for informational purposes only.
