# Supplementary material for: Pharmacist Prescribing as a Strategy to Facilitate Vaccine Accessibility in Ontario, Canada
Source: Pharmacy (Basel). 2026 Aug 25;14(6):125. doi: 10.3390/pharmacy14060125 (PMC13610780; doi:10.3390/pharmacy14060125)
Supplement: Supplementary file 1 [file pharmacy-14-00125-s001.zip › Figure S2 r1.pdf]

EVIDENCE BRIEF

Vaccine accessibility from pharmacies: A Canadian environmental scan

UNIVERSITY OF WATERLOO | SCHOOL OF PHARMACY

Since 2007, pharmacists nationwide have been increasingly granted the authority to administer and prescribe vaccines. **Ontario is currently among the most restrictive provinces related to scope of practice for pharmacists' vaccination services.[1]**

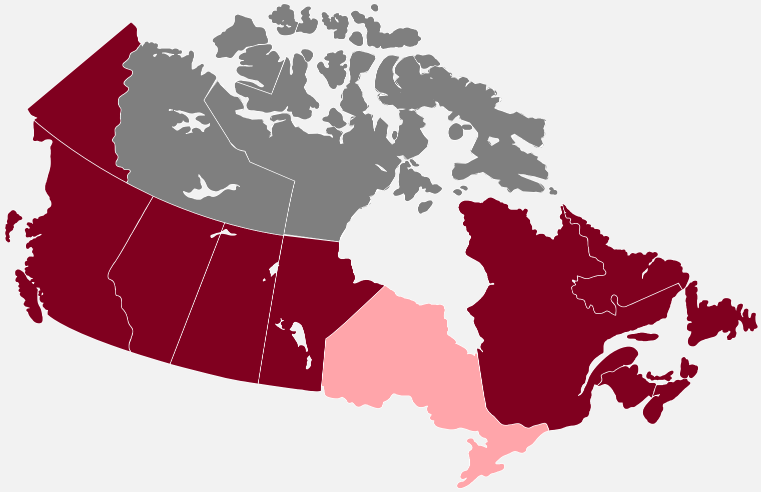

Vaccine Administration Authority

- Broad authority for most/all vaccines
- Administration authorization only for vaccines on a legislated list (those not listed can only be administered with an order from a prescriber)[2]
- No vaccine administration authority

Vaccine Prescribing Authority

- Broad authority for most/all vaccines for routine use (additional credentials may be required for select travel vaccines)
- Prescribing authority available for pharmacists with specific credentials
- No prescribing authority

Additional information on pharmacist vaccine prescribing authority on reverse side

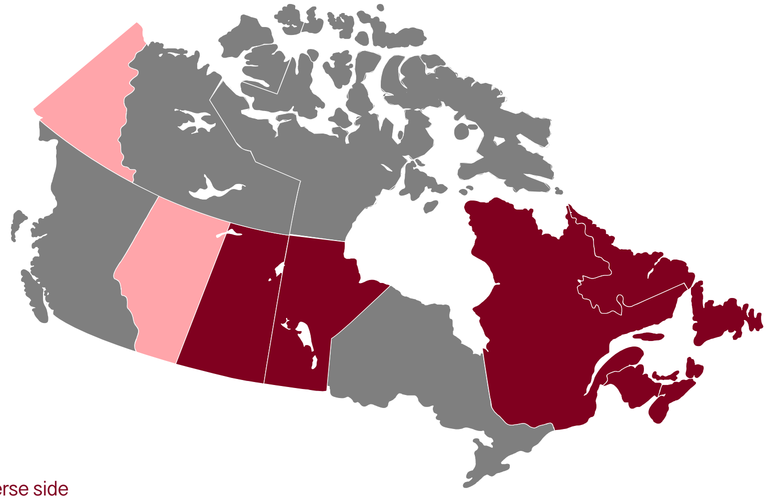

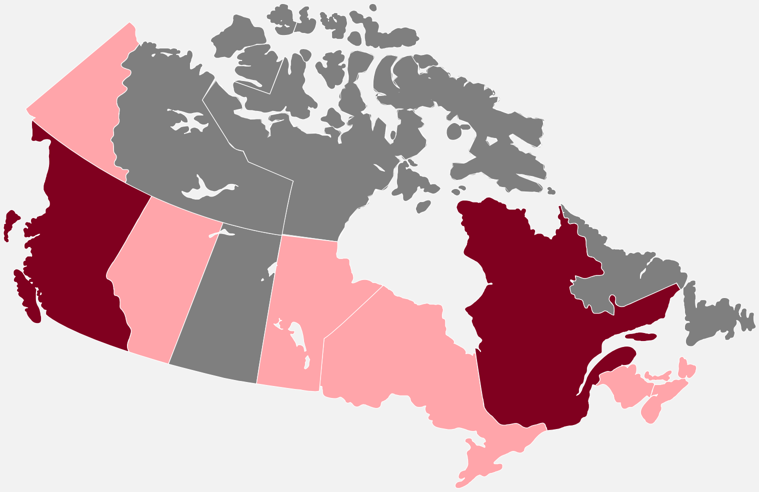

Pharmacy Access to Publicly Funded Vaccines

- Access to most/all publicly funded vaccines
- Access to a selection of publicly funded vaccines **other than** influenza and COVID-19
- No access to publicly funded vaccines **other than** influenza and COVID-19, or no vaccine administration authority

Funding for this project was provided by Merck Canada Inc. Merck Canada Inc. had no role in the development of the content, analysis, conclusions, or recommendations presented herein. The content reflects the independent views of the authors and is intended for informational purposes only.

Vaccine accessibility from pharmacies: A Canadian environmental scan

UNIVERSITY OF WATERLOO | SCHOOL OF PHARMACY

Vaccine prescribing authorization for pharmacists in Canada[3-16]

Province or Territory	Year Implemented	Prescribing Authorization Description
Alberta	2007	• Only pharmacists with Additional Prescribing Authorization credential can prescribe any vaccine
British Columbia	2009	• All publicly funded vaccines as well as any other vaccines recommended by any recognizable provincial, national, or organizational organization are available without a prescription<br>• Pharmacists can not issue prescriptions for others, or if a prescription is required for insurance coverage purposes
Manitoba	2014	• No prescription required for a selection of vaccines to people who are eligible for them through the publicly funded vaccination program<br>• No prescribing authorization for other vaccines or for people ineligible for publicly funded vaccines
New Brunswick	2014	• All pharmacists who complete an orientation training program can prescribe a selection of vaccines<br>• Pharmacists with travel medicine credential can prescribe additional travel vaccines
Newfoundland and Labrador	2024	• All pharmacists who complete an orientation training program can prescribe a selection of vaccines
Nova Scotia	2011	• All pharmacists can prescribe a selection of vaccines<br>• Pharmacists with travel medicine credential can prescribe additional travel vaccines
Prince Edward Island	2020	• All pharmacists who have completed injection training can prescribe a selection of vaccines<br>• Pharmacists with training in travel medicine can prescribe additional travel vaccines
Quebec	2019	• All pharmacists have prescribing authority for all vaccines
Saskatchewan	2019	• All vaccines other than influenza require a prescription; however, all pharmacists can issue prescriptions for non-travel vaccines<br>• Pharmacists with travel medicine credential can prescribe additional travel vaccines

The remaining provinces and territories do not have any vaccine prescribing authorization for pharmacists.

References:

1. **Injection Authority and Vaccine Administration in Pharmacies Across Canada.** Canadian Pharmacists Association. https://www.pharmacists.ca/cpha-ca/assets/File/cpha-on-the-issues/Scope-of-Practice-Immunization_EN.pdf
2. **Administering Injections.** Ontario College of Pharmacists. https://ocpinfo.com/wp-content/uploads/2025/05/administering-injections.pdf
3. **Standards of practice for pharmacists and pharmacy technicians.** Alberta College of Pharmacy. https://abpharmacy.ca/wp-content/uploads/Standards_SPPPT.pdf
4. **2024-25 Annual report.** Alberta College of Pharmacy. https://abpharmacy.ca/flipbook/2024-25-annual-report/
5. **Pharmacists and publicly funded vaccines in B.C.: General information.** BC Ministry of Health. https://www2.gov.bc.ca/assets/gov/health/health-drug-coverage/pharmacare/vaccine-guide.pdf
6. **Administration of injections: Frequently asked questions.** College of Pharmacists of Manitoba. https://cphm.ca/wp-content/uploads/Resource-Library/Expanded-Scope/Administration-of-Injections-Reference-July-2025.pdf
7. **Common ailments and certain conditions.** New Brunswick College of Pharmacists. https://nbpharmacists.ca/en/page_category/legislation/
8. **Travel medicine: Prescribing for preventable diseases.** New Brunswick College of Pharmacists. https://nbpharmacists.ca/wp-content/uploads/2022/11/Travel-Medicine-_Prescribing-of-Restricted-Vaccines_Regs-Appendix3_2014-3.pdf
9. **Standards of practice: Prescribing by pharmacists.** College of Pharmacy of Newfoundland and Labrador. https://cpnl.ca/media/SOP-Prescribing-by-Pharmacists-2023-10.pdf
10. **Prescribing standards of practice.** College of Pharmacy of Newfoundland and Labrador. https://cpnl.ca/professional-practice/area-of-practice/prescribing/
11. **Standards of practice: Prescribing drugs.** Nova Scotia Pharmacy Regulator. https://nspharmacy.ca/wp-content/uploads/SOP_PrescribingDrugs_May2024.pdf
12. **Regulated Health Professions Act: Pharmacist and pharmacy technician regulations.**Prince Edward Island Government. https://www.princeedwardisland.ca/sites/default/files/legislation/r10-1-07-regulated_health_professions_act_pharmacist_and_pharmacy_technician_regulations.pdf
13. **Application requirements – Extended Practice Authorization in Drug Administration.** Prince Edward Island College of Pharmacy. https://pepharmacists.ca/wp-content/uploads/2025/08/RP07-Application-Requirements-EPC-DA-MAY-2025.pdf
14. **Application requirements – Extended Practice Certificate: Travel Vaccine Authority.** Prince Edward Island College of Pharmacy. https://pepharmacists.ca/wp-content/uploads/2023/11/RP10-Application-Requirements-EPC-Travel-Approved-Sept-2023.pdf
15. **La vaccination par le pharmacien: Guide d'exercice.** Ordre des Pharmaciens du Quebec. https://www.opq.org/wp-content/uploads/2020/03/Guide_vaccination_juil2023.pdf
16. **Travel health services and vaccine-preventable diseases FAQs.** Saskatchewan College of Pharmacy Professionals. https://scp.in1touch.org/document/5581/REF_Travel_Health_FAQs.pdf

Author

Sherilyn Houle, PhD
School of Pharmacy, University of Waterloo

https://uwaterloo.ca/pharmacy/profile/shoule
May 2026

Funding for this project was provided by Merck Canada Inc. Merck Canada Inc. had no role in the development of the content, analysis, conclusions, or recommendations presented herein. The content reflects the independent views of the authors and is intended for informational purposes only.
