# Supplementary material for: Pharmacist Prescribing as a Strategy to Facilitate Vaccine Accessibility in Ontario, Canada
Source: Pharmacy (Basel). 2026 Aug 25;14(6):125. doi: 10.3390/pharmacy14060125 (PMC13610780; doi:10.3390/pharmacy14060125)
Supplement: Supplementary file 1 [file pharmacy-14-00125-s001.zip › Figure S3.pdf]

EVIDENCE BRIEF

The societal impact of pharmacist prescribing of vaccines

UNIVERSITY OF WATERLOO | SCHOOL OF PHARMACY

Having to obtain a prescription to be dispensed a vaccine, to be administered a vaccine at a pharmacy, and/or for coverage by a third-party insurance plan, **makes vaccination less convenient and less accessible for Ontarians.**

(see figure on reverse)

Among Ontarians who received a vaccine review (VaxCheck®) by a pharmacist, an average of 3 vaccines were recommended per patient. Many patients interested in vaccination had to delay it as they awaited a prescription:[1]

- **67%** of those recommended hepatitis A vaccine
- **56%** of those recommended hepatitis B vaccine
- **23%** of those recommended pneumococcal vaccine
- **16%** of those recommended herpes zoster (shingles) vaccine

2.5 million Ontarians do not have a family doctor to access a vaccine prescription from[2]

Flexible, community-based, and convenient healthcare settings are valued[3]

Important factors when choosing a site for routine immunization (n=9,055 Canadian respondents)

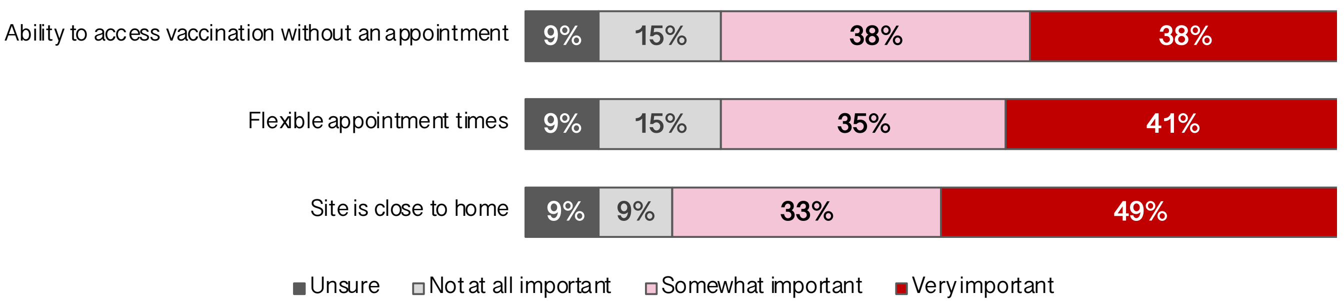

Community pharmacies have unmatched accessibility

89% of Ontario's community pharmacies are open on Saturdays and 50% on Sundays[4]

99.4% of urban residents and 41% of rural residents of Ontario live within 5 km of a pharmacy[5]

Of >1.2 million vaccine appointments scheduled at Canadian pharmacies, 27% were on evenings, weekends, or holidays[6]

Authorizing pharmacists to prescribe vaccines can **promote uptake at the point of recommendation, reduce delays and prevent missed vaccination opportunities, and reduce societal impacts from lost productivity** to access other care providers for a prescription.

Funding for this project was provided by Merck Canada Inc. Merck Canada Inc. had no role in the development of the content, analysis, conclusions, or recommendations presented herein. The content reflects the independent views of the authors and is intended for informational purposes only.

EVIDENCE BRIEF

The societal impact of pharmacist prescribing of vaccines

UNIVERSITY OF WATERLOO | SCHOOL OF PHARMACY

The patient journey to access vaccinations through pharmacies in Ontario, other than for those eligible for publicly-funded vaccines available to pharmacies *

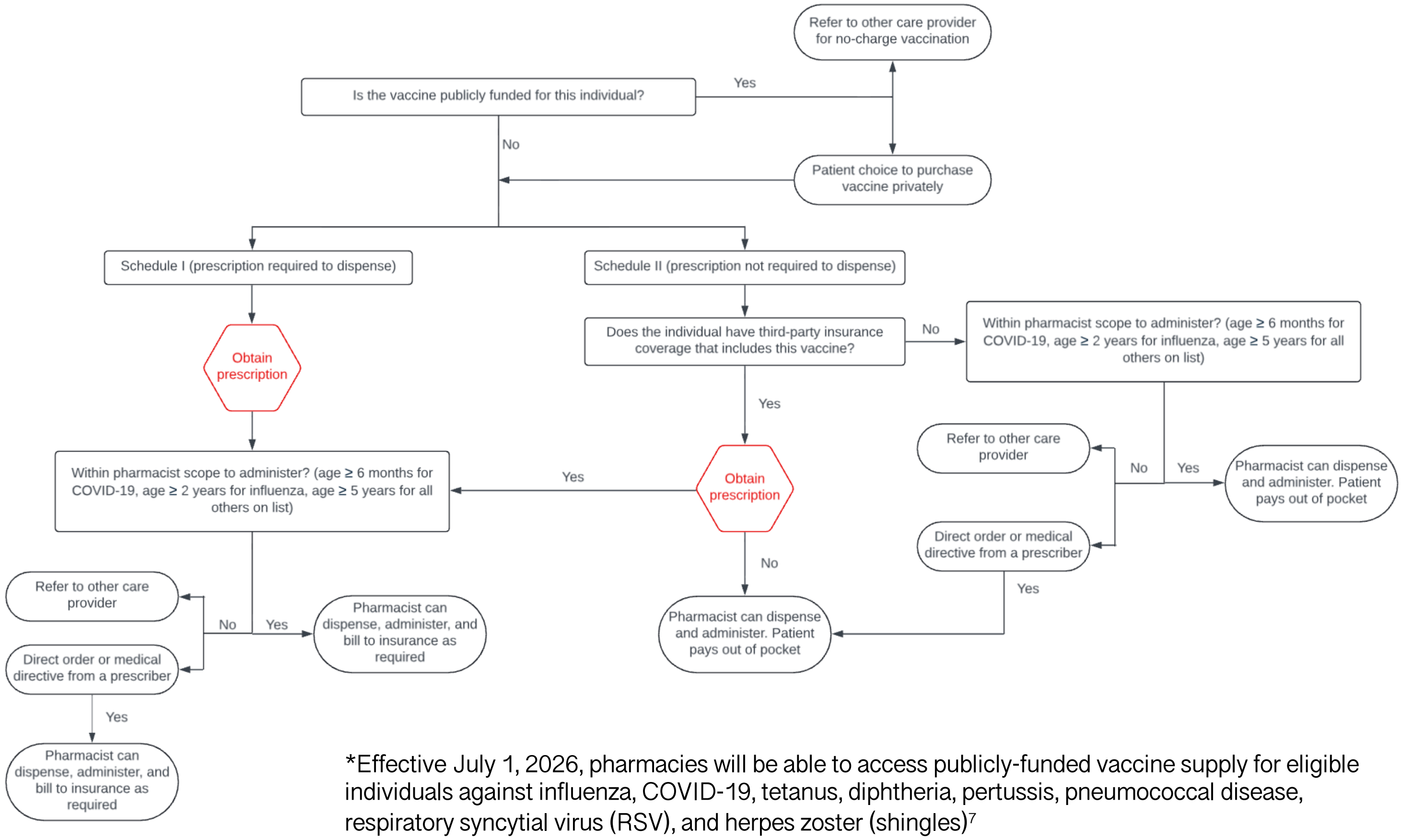

*Effective July 1, 2026, pharmacies will be able to access publicly-funded vaccine supply for eligible individuals against influenza, COVID-19, tetanus, diphtheria, pertussis, pneumococcal disease, respiratory syncytial virus (RSV), and herpes zoster (shingles)[7]

References:

1. Houle SKD, Waite NM, Vernon-Wilson E, Dolovich L, Yang M. **Uptake and outcomes of VaxCheck, an adult life-course vaccination service: A study among community pharmacists.** Vaccine 2024;42(20):126096.
2. Ontario College of Family Physicians. **New data shows there are now 2.5 million Ontarians without a family doctor.** https://ontariofamilyphysicians.ca/news/new-data-shows-there-are-now-2-5-millionontarians-without-a-family-doctor/
3. Neighbourhood Pharmacy Association of Canada and 19toZero. **Routine Immunizations in Canada Following the COVID-19 Pandemic.** https://neighbourhoodpharmacies.ca/sites/default/files/2021-10/Routine%20Immunization%20Final%20Results%20-%20Public%20Slides%20-%2020Oct2021%20vF_0.pdf
4. Statistics Canada. **Measuring proximity to services and amenities: An experimental set of indicators for neighbourhoods and localities**. https://www150.statcan.gc.ca/n1/pub/18-001-s/18-001-x2020001-eng.htm
5. Timony P, Houle SKD, Gauthier A, Waite NM. **Geographic distribution of Ontario pharmacists: A focus on rural and northern communities**. Can Pharm J 2022;155(5):267-276.
6. Houle SKD, Bhaidani S, Rehmanji T, Alsabbagh MW. **Administration of vaccines and injectable medications beyond 9 to 5: A cross-sectional analysis of over 1.2 million injection appointments scheduled at Canadian community pharmacies**. J Am Pharm Assoc 2026;66(3):103079.
7. Ontario Ministry of Health. **Ontario Expanding Scope of Practice for Pharmacists and Other Health Professionals.** Ontario Ministry of Health. https://news.ontario.ca/en/release/1007431/ontario-expanding-scope-of-practice-for-pharmacists-and-other-health-professionals

Author

Sherilyn Houle, PhD
School of Pharmacy, University of Waterloo

https://uwaterloo.ca/pharmacy/profile/shoule

May 2026

Funding for this project was provided by Merck Canada Inc. Merck Canada Inc. had no role in the development of the content, analysis, conclusions, or recommendations presented herein. The content reflects the independent views of the authors and is intended for informational purposes only.
